# Supplementary material for: The Effect of Intravenous Tranexamic Acid on Perioperative Blood Loss, Transfusion Requirements, Verticalization, and Ambulation in Total Knee Arthroplasty: A Randomized Double-Blind Study
Source: Medicina (Kaunas). 2024 Jul 21;60(7):1183. doi: 10.3390/medicina60071183 (PMC11279079; doi:10.3390/medicina60071183)
Supplement: Supplementary file 1 [file medicina-60-01183-s001.zip › medicina-3092473-supplementary.pdf]

Table S1. Descriptive data on intraoperative, postoperative and perioperative blood loss (ml) and results of group comparations (Man-Witney Test)

Blood Loss	Group	Minimum	Maximum	Mean	SD	Median	Interquartile Rang	Skewness	Kurtosis	Z	*p*
**Intraoperative blood loss**	TXA group	0.00	400.00	102.17	93.521	100.00	162.5	0.874	0.807	-6.931	<0.001
	Control group	100.00	1400.00	438.84	287.677	400.00	350.00	1.249	1.672		
**Postoperative blood loss after 6h**	TXA group	50.00	500.00	123.91	75.821	100.00	50.00	2.802	12.604	-4.511	<0.001
	Control group	100.00	600.00	203.72	100.120	200.00	150.00	1.555	4.240		
**Postoperative blood loss after 12h**	TXA group	0.00	300.00	90.22	82.744	100.00	162.5	0.603	-0.581	-1.749	0.080
	Control group	0.00	550.00	131.40	114.966	100.00	150.00	1.282	2.787		
**Postoperative blood loss after 24h**	TXA group	0.00	400.00	95.65	97.083	100.00	150.00	1.194	1.386	-1.240	0.215
	Control group	0.00	300.00	113.95	86.138	100.00	150.00	0.658	-0.336		
**Total postoperative blood loss**	TXA group	50.00	850.00	309.78	143.612	300.00	125.00	1.283	3.566	-4.319	<0.001
	Control group	100.00	1350.00	449.07	196.309	450.00	150.00	2.274	9.786		
**Perioperative blood loss**	TXA group	150.00	850.00	411.96	172.738	375.00	200.00	0.812	0.289	-6.512	<0.001
	Control group	450.00	1700.00	892.56	324.456	800.00	530.00	0.652	-0.499		

Table S2. Descriptive data about postoperative recovery and distribution analysis (Chi-Square ($\chi 2$) Test)

		**TXA Group**		**Control group**		**$\chi 2$**	***p***
		N	%	N	%		
First meal	Day zero	40	83.3	29	60.4	5.942	0.022
	Day 1	8	16.7	19	39.6		
Standing	Day zero	28	58.3	6	12.5	21.162	<0.001
	Day 1	20	41.7	42	87.5		
Ambulation	Day zero	24	50,0	2	4.2	26,274	<0.001
	Day 1	24	50.0	46	95.8		
	Day 2	0	0.0	3	6.3		
